# Correction to: Changes in patterns of eating habits and food intake during the first German COVID-19 lockdown: results of a cross-sectional online survey

**DOI:** 10.1007/s00394-022-02952-6

**Published:** 2022-07-14

**Authors:** Judith Bühlmeier, Stefanie Frölich, Christine Ludwig, Nadja Knoll-Pientka, Börge Schmidt, Manuel Föcker, Lars Libuda

**Affiliations:** 1 Department of Child and Adolescent Psychiatry, University Hospital Essen, University of Duisburg-Essen, Essen, Germany; 2 Faculty of Natural Sciences, Institute of Nutrition, Consumption and Health, Paderborn University, Paderborn, Germany; 3 Institute for Medical Informatics, Biometry and Epidemiology, University Hospital Essen, University of Duisburg-Essen, Essen, Germany; 4 LVR Clinic for Psychosomatic Medicine and Psychotherapy, University of Duisburg-Essen, University Hospital Essen, Essen, Germany; 5 Department of Child and Adolescent Psychiatry, University Hospital Münster, Münster, Germany; 6 Evangelisches Krankenhaus Düsseldorf, Children’s Hospital, Düsseldorf, Germany

## Correction to: European Journal of Nutrition 10.1007/s00394-022-02919-7

The original version of this article unfortunately contained a mistake. Figures 1 and 2 were swapped incorrectly.

The correct Figs. 1 and 2 are placed in the following page.

**Fig. 1:**
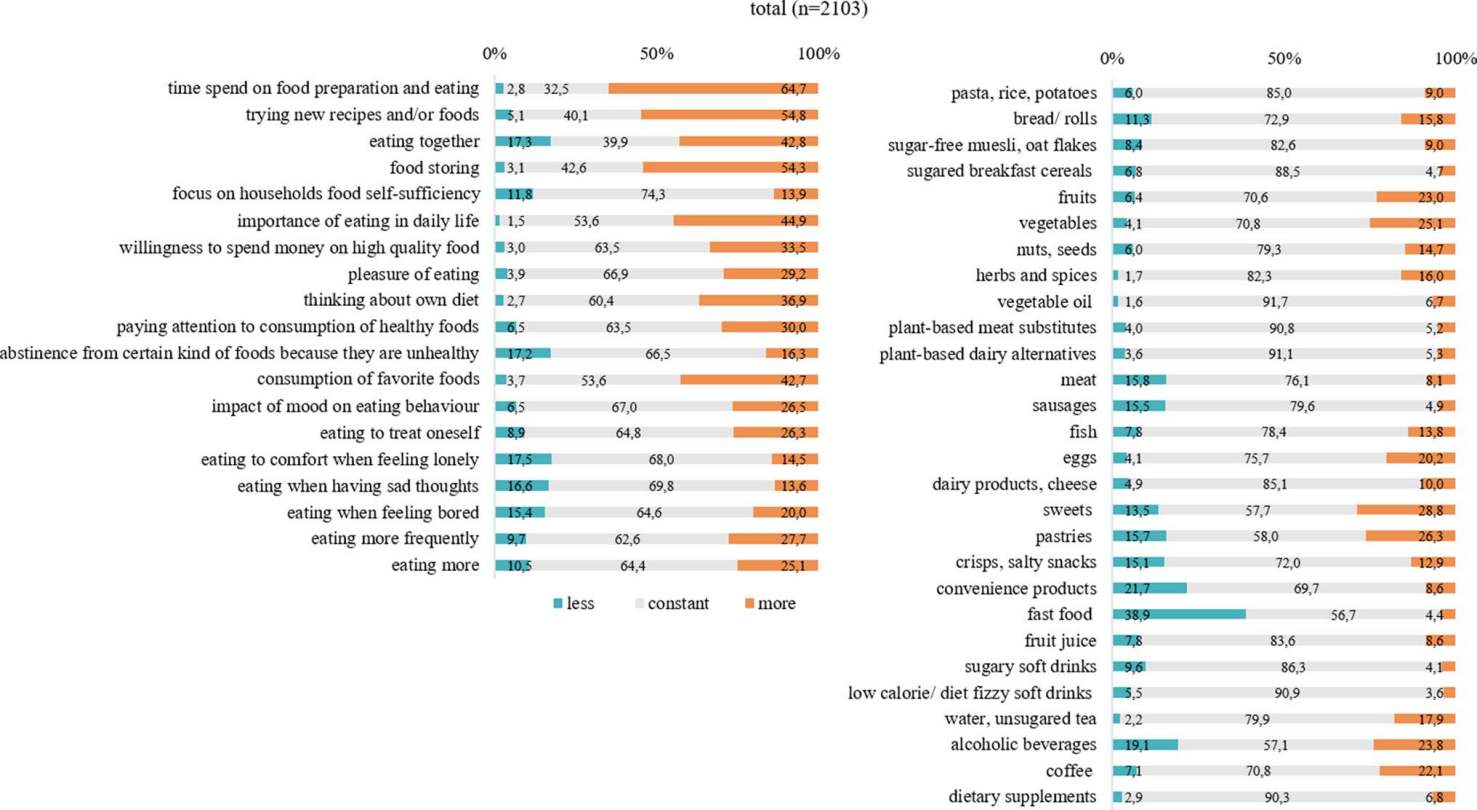
Changes in eating habits and food intake (total sample)

**Fig. 2:**
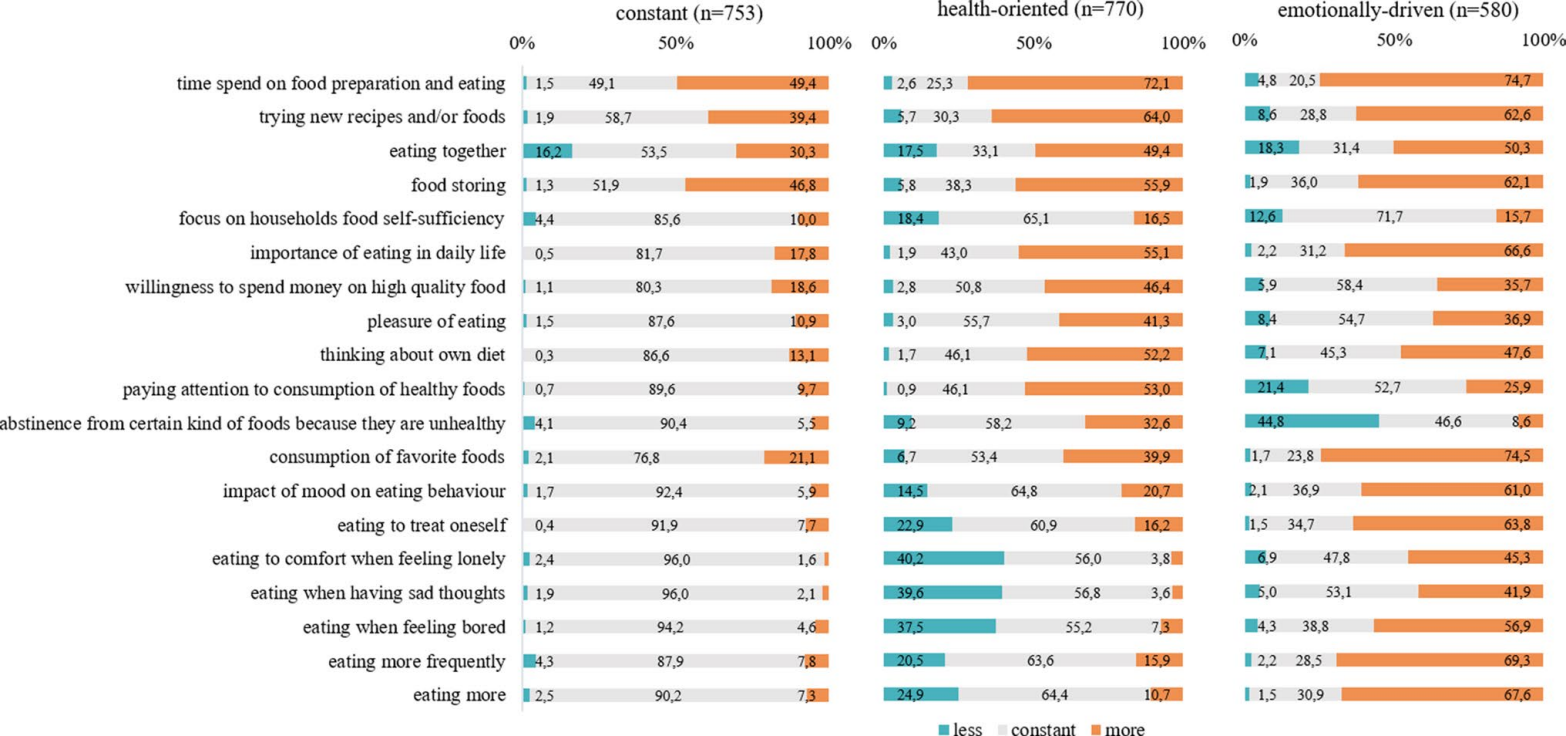
Changes in eating habits according to patterns of change

The original article has been corrected.

